# Supplementary material for: Surface Microbiome Profiling of Dental Elevators Using Third‐Generation Sequencing: Implications for Infection Control in Dental Practice
Source: Microbiologyopen. 2025 Dec 8;14(6):e70178. doi: 10.1002/mbo3.70178 (PMC12685768; doi:10.1002/mbo3.70178)
Supplement: Supplementary file 1 — customer_backup. [file MBO3-14-e70178-s001.zip › customer_backup/customer_backup/taxa_summary/krona/groups/treat/M12.Krona.html]

Javascript must be enabled to view this page.

members
magnitude
magnitudeUnassigned

M12.krona

57524

57516

63

0

0

0

0

0

0

0

0

0

0

0

0

0

0

0

63

63

63

63

10

7

7

0

7

11

13

0

8

0

0

0

0

0

0

0

0

0

0

0

0

0

0

356

304

1

1

1

1

3

3

0

0

2

2

0

0

0

1

1

0

0

300

4

4

4

1

0

0

1

1

1

1

1

0

0

0

0

0

2

0

0

0

0

0

0

2

0

2

292

292

292

0

0

52

17

0

0

0

17

17

17

35

10

10

10

4

4

4

2

2

2

5

5

5

14

14

14

72

65

65

0

0

0

65

40

40

0

18

4

14

0

0

7

7

0

0

0

0

0

0

0

0

0

0

0

0

0

0

0

0

0

0

0

0

0

0

0

0

0

0

0

0

0

0

0

0

0

0

0

0

0

0

0

0

0

0

0

0

0

0

0

0

0

0

3

3

1

0

0

0

0

0

0

0

0

0

0

1

1

0

0

0

0

0

0

0

0

0

0

0

2

0

0

0

0

0

2

2

4

1

1

0

0

1

1

0

0

0

0

0

0

0

0

0

0

0

0

0

3

3

3

3

0

0

0

0

0

0

0

0

0

0

0

0

0

0

0

2

2

1

1

1

1

0

0

0

0

1

1

1

1

8

8

8

0

0

0

4

4

4

1

1

1

0

0

0

3

3

3

551

121

121

121

7

7

72

72

37

1

36

5

4

1

14

14

14

14

14

416

416

416

89

87

2

316

0

8

166

8

134

7

7

4

3

1

389

120

120

0

0

0

120

120

120

1

1

1

1

1

0

0

0

0

0

0

0

0

0

0

0

268

143

129

80

26

46

8

49

3

25

21

0

14

14

14

0

5

0

0

0

5

2

2

3

3

112

26

5

5

1

1

20

4

16

0

0

0

0

0

0

0

30

0

0

0

30

5

25

0

0

56

56

13

43

8

8

3

3

0

0

1

1

4

1

3

0

0

0

0

0

0

0

33046

14683

0

0

0

0

475

36

36

36

348

3

0

3

27

26

1

10

10

308

1

306

1

91

91

91

232

232

154

1

153

74

74

4

4

23

0

0

0

23

4

4

0

0

0

0

19

19

0

0

0

55

55

24

2

1

21

31

3

4

0

24

2

1

1

1

1

1

1

4439

4439

2

2

232

24

9

89

8

102

0

0

88

88

2

2

119

119

6

6

0

0

0

0

19

5

0

5

4

0

1

4

0

0

586

5

372

65

144

0

0

0

3290

0

0

0

67

3

9

1

0

1

14

1

7

298

62

1

0

6

927

685

1

1018

0

2

27

151

1

4

0

2

2

95

11

2

0

82

22

21

21

21

1

1

1

0

0

7746

212

16

16

44

36

8

8

8

0

0

0

17

13

4

127

42

9

76

14

14

14

335

21

0

21

211

47

82

7

3

5

62

0

5

103

103

1

1

1

4

4

4

4

4

4

5

5

5

6

6

6

4676

0

0

5

5

0

0

0

283

276

7

185

179

6

2693

2693

0

0

0

0

0

37

37

1071

20

141

822

29

8

1

50

13

13

367

3

39

6

27

4

5

7

4

1

32

15

224

22

22

135

3

3

4

4

102

102

12

12

14

14

0

0

0

11

11

11

2

2

2

9

9

9

121

9

1

2

6

0

0

0

33

33

79

5

37

13

24

67

55

54

1

12

12

0

535

18

5

13

36

36

114

114

39

34

5

2

2

34

34

18

18

5

5

269

54

215

32

32

0

32

1577

377

377

15

15

5

5

33

27

1

5

0

0

570

24

46

7

0

178

37

103

62

9

11

83

10

0

0

21

21

21

8

2

9

2

0

0

2

2

3

3

530

530

0

0

0

0

0

0

242

7

3

3

4

4

235

120

8

2

61

49

31

4

27

20

5

2

13

0

14

14

50

1

49

0

13

13

13

13

26

26

20

0

0

20

6

0

6

0

0

0

3

3

3

3

0

0

0

0

3

3

3

3

74

74

74

71

3

3

3

3

3

0

0

0

0

0

0

0

3

0

0

0

3

3

3

154

154

1

1

39

4

35

0

0

26

11

4

3

5

0

1

2

0

2

2

27

10

17

0

0

0

58

17

11

30

0

0

1

1

0

0

0

0

297

14

14

14

0

0

0

12

12

12

5

5

5

168

168

168

2

2

2

1

1

1

8

8

8

87

87

87

871

871

0

0

12

12

0

0

0

0

44

44

0

0

0

0

0

0

0

0

0

0

0

0

0

5

5

44

44

0

0

0

0

0

22

8

3

7

3

1

727

18

278

431

0

0

1

1

16

4

12

0

18363

0

0

0

0

0

12

12

0

0

12

4

8

15

14

14

14

1

1

1

11204

104

0

0

104

104

34

0

0

7

7

27

27

5

1

1

0

0

0

4

4

0

0

0

0

2

2

2

9

9

9

64

64

64

0

0

6656

1

1

1

1

5224

5224

535

13

4

60

458

895

2

64

8

5

29

430

34

48

11

112

1

10

41

47

2

51

126

1

1

17

17

75

0

69

6

15

15

1

1

17

0

17

7

7

7

70

1

1

2

2

1

1

53

1

2

50

13

10

3

0

0

0

0

0

0

0

0

0

0

0

0

0

0

0

117

117

117

3955

0

0

24

1

4

19

11

11

523

523

44

44

151

114

10

27

604

122

482

4

4

6

0

6

0

0

84

1

83

161

41

120

0

5

5

2121

0

2121

216

216

1

1

55

24

24

31

1

30

61

58

34

24

10

20

20

4

4

3

3

0

3

0

0

0

0

9

9

9

9

1

1

1

1

0

0

0

0

0

0

0

0

3

3

3

3

2

2

2

2

0

0

0

0

0

0

0

0

0

0

0

2109

2

2

2

55

7

6

1

1

1

0

0

5

5

42

0

2

1

39

0

0

0

0

0

0

0

0

0

0

0

0

0

0

0

0

0

0

0

0

0

2051

2051

20

0

1948

83

1

0

0

0

0

0

0

0

0

0

0

0

1

1

0

0

0

0

0

5

5

5

5

0

0

0

0

13

13

0

0

0

0

13

13

0

0

0

0

0

0

0

0

0

0

0

0

0

0

0

0

0

0

0

0

0

0

0

1630

1

1

1

0

0

0

0

0

1

0

0

0

1

1

0

0

0

1125

373

373

8

8

131

131

613

613

3

3

1

2

377

377

377

114

114

114

9

9

9

0

0

0

0

0

0

0

0

0

0

0

0

0

0

0

0

0

0

0

0

0

0

0

0

0

0

0

0

109

109

109

6

3

2

75

9

8

0

6

0

0

0

0

0

0

0

0

0

0

0

3070

544

193

193

51

1

50

294

294

2

2

4

1

3

2526

855

855

6

2

1

3

19

14

5

30

0

13

13

4

1616

0

0

477

1

6

0

474

131

33

8

29

42

415

3

0

0

0

3

3

3

0

0

0

0

0

0

0

0

0

0

0

0

0

0

0

47

47

40

7

14

19

0

0

4

4

0

0

0

0

0

0

0

0

3

3

0

0

0

0

4

4

4

4

59

0

0

0

0

0

0

59

59

59

1

1

1

0

1

0

0

6

6

6

6

0

430

295

295

1

1

1

294

13

13

12

12

21

21

41

41

29

10

19

34

34

0

0

144

101

33

10

135

0

0

0

0

0

0

0

3

2

2

2

0

0

0

1

1

1

78

27

1

1

0

0

0

0

3

1

2

23

7

3

13

2

2

2

0

0

0

0

35

2

1

1

33

0

14

11

8

14

2

2

12

12

0

0

0

0

45

45

45

0

3

19

23

0

0

0

0

0

0

0

0

0

0

0

0

0

0

0

9

9

0

0

9

9

26

26

26

26

26

0

3

4

19

0

0

0

0

0

0

3298

0

0

0

0

0

3286

966

4

4

4

936

11

10

1

12

3

5

4

25

6

11

6

2

27

4

21

2

5

5

2

2

417

4

25

13

10

16

328

5

5

11

149

1

79

60

5

4

3

3

0

0

26

12

3

11

90

90

0

0

102

35

67

0

67

67

26

26

1

14

11

0

0

0

688

0

0

0

2

0

0

0

0

0

0

0

0

0

0

0

0

0

0

0

0

0

0

2

0

2

0

0

0

0

0

0

0

0

0

0

0

0

0

0

0

0

0

0

0

0

0

0

0

0

0

0

0

0

0

0

0

0

0

686

0

0

0

0

686

686

0

0

1389

0

0

0

0

0

0

0

0

0

0

0

440

12

12

30

30

12

12

386

386

41

0

0

0

0

0

0

0

0

0

0

0

0

0

41

2

11

14

1

2

3

0

4

4

0

0

0

0

40

1

1

0

0

39

3

2

34

0

0

0

0

0

0

0

0

381

381

1

0

0

75

169

2

1

0

1

0

54

4

48

5

1

0

20

180

28

28

0

0

0

152

50

31

22

24

0

25

307

0

0

0

16

0

16

241

0

0

241

0

0

50

50

0

0

0

0

0

0

0

0

0

0

0

0

0

0

0

0

0

0

0

0

0

0

0

0

0

0

0

0

0

0

0

0

0

0

0

0

0

0

0

0

0

0

0

0

0

0

0

0

0

0

0

0

0

0

0

0

0

0

0

11

0

0

0

11

7

1

6

0

4

4

0

0

0

0

0

0

0

0

0

0

0

0

0

0

0

232

16

0

0

0

0

2

2

14

1

9

4

6

2

2

3

0

3

0

0

0

1

1

9

0

0

2

2

5

3

2

0

0

2

2

0

3

3

3

55

41

41

8

8

6

2

3

1

143

7

7

60

0

7

2

37

4

10

4

4

0

0

0

11

6

5

61

1

12

1

3

32

2

2

0

0

1

5

0

2

0

0

0

0

0

4

4

4

4

4

0

0

8

8

0

0

0

8

8

8

0

0

0

0

0

0

0

0

0

0

0

0

0

0

0

187

142

2

2

0

0

0

0

0

2

0

2

0

0

0

0

0

0

0

0

0

0

0

0

0

0

0

0

0

140

139

139

139

1

1

1

0

0

0

0

45

38

38

19

17

2

4

4

15

5

10

7

7

7

7

0

0

0

0

0

0

0

0

7861

106

106

1

1

1

3

3

0

3

0

0

0

0

0

0

0

102

0

0

7

0

4

2

1

2

2

19

19

6

5

1

0

0

1

1

0

44

0

42

2

23

1

20

2

0

0

76

76

76

76

76

1148

69

69

0

0

0

0

48

48

5

5

12

12

4

4

0

0

0

0

0

0

168

25

25

25

21

21

21

5

5

5

7

7

7

7

7

7

103

103

103

251

7

7

7

3

3

3

40

40

40

2

2

2

173

173

173

26

26

26

2

2

2

2

658

46

3

0

3

43

0

43

0

0

0

0

146

146

1

7

138

220

15

1

14

197

2

195

8

5

3

246

0

0

5

5

241

241

2413

1465

3

3

3

661

480

1

169

5

47

85

131

42

1

1

16

11

5

82

4

2

76

50

21

17

12

32

32

801

55

55

3

3

739

739

0

0

4

4

948

453

453

50

396

7

495

0

0

4

4

491

491

4052

4

4

4

4

1

1

1

1

0

0

0

0

0

0

0

0

3

3

3

3

43

43

3

3

1

1

4

2

2

1

1

3

3

2

2

5

2

1

0

2

10

0

8

2

3

3

9

9

0

0

2

2

15

15

13

0

1

12

0

0

2

2

150

1

0

0

0

0

0

1

1

61

61

6

29

19

7

88

78

2

76

0

0

7

7

3

3

39

39

0

0

0

39

0

18

8

0

0

2

0

11

0

0

223

97

19

19

77

77

1

1

20

0

0

20

20

2

2

0

2

3

3

3

4

4

0

4

97

3

3

17

17

77

77

16

1

0

0

0

1

1

15

0

0

15

13

2

1

1

1

1

0

0

0

0

0

0

0

0

0

0

0

0

0

0

0

0

9

9

9

9

0

0

0

43

43

0

0

43

15

5

9

0

13

0

1

484

81

19

19

0

0

0

0

0

0

0

0

0

0

0

2

1

1

8

0

8

0

52

52

403

28

7

8

13

4

4

329

0

1

10

1

0

1

107

11

10

3

23

20

9

1

59

1

1

0

6

49

2

7

7

16

16

24

0

24

2

2

78

78

0

0

1

1

0

0

0

0

0

1

1

49

0

8

6

6

19

10

12

2

0

1

9

4

4

6

6

5

5

2943

2145

4

4

1

1

270

0

270

0

0

25

25

0

1516

4

1025

19

366

102

1

1

2

2

326

326

42

4

4

1

1

3

3

5

0

5

25

3

22

4

4

0

0

0

0

0

172

155

152

1

2

17

17

548

1

1

0

0

10

10

12

2

10

0

0

0

0

1

1

13

13

3

3

3

3

0

0

11

11

481

0

50

431

9

9

3

3

1

1

0

0

4

4

4

32

23

23

9

9

66

66

66

66

4

30

26

6

5

4

4

4

0

0

0

0

0

0

0

0

0

0

0

0

0

0

0

0

0

0

0

0

0

0

0

0

0

0

4

4

0

0

0

0

0

0

0

1

1

1

1

1

413

413

413

413

413

413

221

221

221

221

220

220

0

0

0

0

1

1

0

0

0

0

0

0

0

0

0

0

0

0

71

71

71

70

70

0

15

55

0

0

0

0

0

0

0

0

0

0

0

0

0

1

1

1

34

0

0

0

0

0

6

6

4

4

4

2

2

2

0

0

28

28

0

0

0

5

5

5

23

23

23

9

9

9

9

8

5

3

1

1

615

310

21

21

21

21

289

92

81

74

7

2

2

1

1

8

8

197

32

32

20

20

7

7

138

138

145

55

55

55

10

38

6

1

4

3

3

3

1

1

1

86

86

1

1

31

0

12

19

27

4

23

24

8

1

15

3

0

0

3

11

0

0

0

0

11

11

11

11

0

0

0

0

76

76

76

0

0

76

3

0

1

0

26

7

19

20

0

0

0

0

0

73

73

6

6

6

55

55

55

12

12

12

0

0

0

0

755

2

0

0

0

0

0

0

0

0

2

2

2

2

16

2

2

2

2

6

1

1

1

5

5

5

1

1

1

1

5

2

2

2

3

3

3

0

0

0

0

2

2

2

2

8

0

0

0

0

0

0

0

0

8

1

1

1

4

4

4

3

3

3

0

0

0

0

0

729

729

502

502

502

35

35

35

0

0

0

18

18

18

0

0

12

12

12

50

0

0

50

0

50

0

0

0

0

0

0

0

0

26

26

26

86

86

86

18

18

18

0

0

0

18

18

18

0

0

0

0

0

0

0

0

0

0

0

0

0

0

0

0

0

0

0

0

101

62

12

12

4

4

8

6

2

0

0

0

0

50

21

21

21

18

18

18

0

0

0

1

1

1

7

7

7

3

3

3

18

18

18

18

18

21

13

13

7

7

6

0

6

8

8

8

1

7

38

38

38

15

15

0

15

0

23

11

4

7

12

1

0

0

0

0

2

9

4

4

4

4

0

0

4

4

8598

4209

7

7

3

2

1

1

1

3

1

0

0

1

0

1

1

1

0

0

1

1

32

2

2

2

1

1

1

29

29

29

0

0

13

13

13

3

10

0

2453

2453

2

2

0

0

0

0

0

0

0

107

37

70

4

0

4

58

50

0

8

0

0

8

2

6

23

23

0

0

4

4

3

3

0

0

0

0

0

0

31

1

0

30

0

0

86

0

2

2

0

1

1

0

0

0

41

14

2

5

1

0

0

3

4

2

8

0

0

0

0

27

0

27

0

0

51

0

46

5

353

131

9

0

212

0

1

20

1

0

0

5

13

0

1

0

0

13

13

241

19

0

0

214

8

85

85

0

0

0

0

0

0

0

3

3

0

0

0

0

0

50

50

5

5

125

0

8

82

35

0

0

3

3

40

40

4

4

5

5

10

6

4

0

0

0

45

16

29

4

4

0

1043

5

463

1

14

1

196

321

0

2

21

19

0

0

0

0

0

0

0

0

0

0

0

578

30

0

0

10

5

5

0

0

0

0

0

0

0

0

0

0

0

16

0

2

0

0

14

4

4

2

2

2

534

4

4

0

0

0

0

0

0

0

0

375

375

155

33

122

12

3

3

4

3

1

4

4

1

1

0

0

0

0

647

647

0

0

0

0

647

3

3

5

0

4

628

4

0

0

17

6

6

6

0

0

0

0

0

0

3

3

3

8

8

8

0

0

0

0

0

1

1

1

1

446

255

9

2

7

1

1

72

72

2

2

0

0

0

0

0

25

0

25

0

0

3

3

0

0

15

10

2

2

1

8

1

1

0

6

0

12

0

1

0

4

0

0

2

5

0

0

0

0

49

49

27

4

23

32

0

0

0

32

51

51

51

0

0

0

18

1

0

1

13

10

0

0

3

4

4

2

2

2

0

0

0

5

2

2

3

3

115

28

3

0

0

3

21

1

8

8

0

0

5

3

2

0

0

0

0

29

13

8

4

4

42

42

0

0

0

3

2

1

0

0

0

14

7

7

7

0

0

0

7

7

7

0

0

0

0

0

0

4332

0

0

0

0

1160

314

0

0

2

2

5

5

30

27

1

1

0

1

58

2

56

161

161

37

37

21

21

0

0

846

0

0

0

0

0

0

0

846

5

0

6

56

7

17

54

10

0

278

370

1

19

0

0

18

5

0

0

0

0

0

0

0

0

0

0

0

0

0

483

155

0

0

0

0

22

22

96

30

0

0

0

57

9

0

0

1

1

26

16

10

10

10

328

13

13

7

7

53

27

26

13

3

10

78

53

25

4

4

0

0

61

61

84

15

69

1

1

8

8

0

0

4

4

2

2

0

0

0

0

1

1

1

1

13

12

3

3

3

3

6

0

6

1

1

1

0

0

5

5

5

5

0

332

332

0

0

1

1

279

49

99

0

0

2

3

0

1

7

21

10

3

0

8

41

0

3

2

2

1

5

15

2

0

3

2

0

0

11

11

41

5

21

15

9

1

1

1

0

0

0

6

6

6

2

2

2

2110

2110

2110

2091

19

215

7

7

6

1

2

1

1

0

0

1

1

0

0

0

0

40

36

0

2

5

0

7

0

18

2

2

0

0

0

0

4

4

0

15

15

0

15

151

0

0

0

0

14

12

1

1

3

0

3

0

43

0

22

3

11

0

7

1

1

20

19

0

0

0

0

1

11

11

39

0

0

39

10

1

9

1

1

9

9

0

0

0

0

0

0

0

0

0

0

0

4

4

4

0

0

0

2

0

2

0

0

0

0

0

0

0

0

0

0

0

0

0

0

0

0

0

0

1

1

1

1

1

32

0

0

0

0

0

0

32

0

0

0

4

0

0

0

0

0

0

3

3

0

1

0

0

0

0

0

0

0

0

1

20

6

6

0

0

0

14

14

0

0

0

0

0

0

8

8

8

24

24

24

24

24

122

14

1

1

1

1

0

0

0

0

0

6

6

6

6

7

5

5

5

2

2

2

0

0

0

3

2

2

2

2

1

0

0

0

1

1

1

0

0

0

0

36

19

19

19

19

2

2

0

0

0

2

2

0

0

0

6

6

6

6

0

0

0

5

5

1

1

4

4

0

0

4

3

3

3

1

1

1

0

0

0

69

69

69

69

69

7

7

7

7

7

3

4

161

161

161

161

161

74

87

0

0

0

0

0

0

55

55

55

6

6

6

10

10

10

31

31

31

8

8

8

0

0

0

0

0

0

0

0

0

0

0

0

0

8

1

1

1

0

0

0

1

1

1

0

0

0

0

0

0

0

0

5

5

5

5

5

5

2

2

2

2

2

2
